# Ultra-High-Resolution Optical Remote Sensing Satellite Identification of Pine-Wood-Nematode-Infected Trees

**DOI:** 10.3390/plants14223436

**Published:** 2025-11-10

**Authors:** Ziqi Nie, Lin Qin, Peng Xing, Xuelian Meng, Xianjin Meng, Kaitong Qin, Changwei Wang

**Affiliations:** 1 College of Natural Resources and Environment, South China Agricultural University, Guangzhou 510642, China; nzq2022331@163.com (Z.N.); kaitong_qin@163.com (K.Q.); 2 State Key Laboratory of Surveying, Mapping and Remote Sensing Information Engineering, Wuhan University, Wuhan 430079, China; 3 Aerial Photogrammetry and Remote Sensing Group Co., Ltd., Xi’an 710199, China; 4 Guangdong Provincial Institute of Forestry Survey and Planning, Guangzhou 510520, China; luckykql@263.net (L.Q.); 18933986718@163.com (X.M.); 5 Guangzhou Institute of Geography, Guangzhou 510070, China; kaiers@126.com; 6 Department of Geography & Anthropology, Louisiana State University, Baton Rouge, LA 70803, USA; smeng@lsu.edu; 7 Guangdong Engineering Technology Research Center of Land Information, Guangzhou 510642, China

**Keywords:** pine wood nematode, Beijing3N remote sensing image, U-NET deep learning, image resolution, number of bands of the image

## Abstract

The pine wood nematode (PWN), one of the globally significant forest diseases, has driven the demand for precise detection methods. Recent advances in satellite remote sensing technology, particularly ultra-high-resolution optical imagery, have opened new avenues for identifying PWN-infected trees. In order to systematically evaluate the ability of ultra-high-resolution optical remote sensing and the influence of spatial and spectral resolution in detecting PWN-infected trees, this study utilized a U-Net network model to identify PWN-infected trees using three remote sensing datasets of the ultra-high-resolution multispectral imagery from Beijing 3 International Cooperative Remote Sensing Satellite (BJ3N), with a panchromatic band spatial resolution of 0.3 m and six multispectral bands at 1.2 m; the high-resolution multispectral imagery from the Beijing 3A satellite (BJ3A), with a panchromatic band resolution of 0.5 m and four multispectral bands at 2 m; and unmanned aerial vehicle (UAV) imagery with five multispectral bands at 0.07 m. Comparison of the identification results demonstrated that (1) UAV multispectral imagery with 0.07 m spatial resolution achieved the highest accuracy, with an F1 score of 89.1%. Next is the fused ultra-high-resolution BJ3N satellite imagery at 0.3 m, with an F1 score of 88.9%. In contrast, BJ3A imagery with a raw spatial resolution of 2 m performed poorly, with an F1 score of only 28%. These results underscore that finer spatial resolution in remote sensing imagery directly enhances the ability to detect subtle canopy changes indicative of PWN infestation. (2) For UAV, BJ3N, and BJ3A imagery, the identification accuracy for PWN-infected trees showed no significant differences across various band combinations at equivalent spatial resolutions. This indicates that spectral resolution plays a secondary role to spatial resolution in detecting PWN-infected trees using ultra-high-resolution optical imagery. (3) The 0.3 m BJ3N satellite imagery exhibits low false-detection and omission rates, with F1 scores comparable to higher-resolution UAV imagery. This indicates that a spatial resolution of 0.3 m is sufficient for identifying PWN-infected trees and is approaching a point of saturation in a subtropical mountain monsoon climate zone. In conclusion, ultra-high-resolution satellite remote sensing, characterized by frequent data revisit cycles, broad spatial coverage, and balanced spatial-spectral performance, provides an optimal remote sensing data source for identifying PWN-infected trees. As such, it is poised to become a cornerstone of future research and practical applications in detecting and managing PWN infestations globally.

## 1. Introduction

The pine wood nematode (PWN, *Bursaphelenchus xylophilus*), the causative agent of pine wilt disease, ranks among the world’s most destructive forest pathogens due to its rapid spread, high mortality rates, and severe economic impacts [1,2]. Since its first detection in Nanjing, China, in 1982, PWN has devastated over one billion pine trees nationwide, incurring losses exceeding hundreds of billions of yuan [3]. Early detection of PWN-infected trees remains critical for containing outbreaks, as timely and accurate identification of PWN-infected trees is important for prevention and control [4].

Traditional methods for identifying PWN-infected trees have relied on labor-intensive field surveys. These approaches lack the capacity for rapid, large-scale monitoring of PWN infestation dynamics and often lead to delayed interventions that hinder effective disease management [1,5,6,7]. Remote sensing technology has emerged as a primary investigative tool for detecting PWN-infected trees due to its cost-effectiveness, operational efficiency, and streamlined data-collection capabilities [8,9,10]. Early identification of PWN-infected trees through satellite remote sensing mainly depended on medium-resolution satellites, such as Landsat, which utilized the spectral differences between the canopies of infected and healthy pine trees [11,12]. Franklin et al. [13] achieved approximately 73% detection accuracy for PWN-infected trees using single-date 30 m resolution Landsat imagery. Skakun et al. [14] enhanced PWN identification accuracy by to 78% by analyzing multi-temporal Landsat data in the Prince George Forest Region of British Columbia, Canada. However, mixed-pixel effects in medium-resolution imagery obscure sparse infections, limiting practical utility.

Advances in high-resolution imagery (≤1 m) have improved detection precision. Wang et al. [15] utilized Gaofen-2 satellite imagery with 1 m spatial resolution and four spectral bands, achieving an MIoU of 68.36%. Poona and Ismail [16] aimed to explore the utility of transformed high spatial resolution QuickBird imagery, which has a 0.6 m spatial resolution and four spectral bands, combined with artificial neural networks (ANNs), to detect and map trees infested with PWN, achieving a kappa coefficient of 0.65. Takenaka et al. [17] employed WorldView-3 imagery with 0.5 m spatial resolution to construct 18 vegetation indices for identifying PWN-infected trees in the Matsumoto region of central Japan, achieving a total precision of 72%. Despite these improvements, resolution thresholds for reliable PWN detection remain undefined, and spectral-spatial tradeoffs are poorly quantified.

The recent deployment of ultra-high-resolution satellites, with a resolution of less than 0.5 m, presents significant potential. The Beijing 3 International Cooperative Remote Sensing Satellite (BJ3N), launched on 29 April 2021, is currently the highest-resolution commercial remote sensing satellite. It offers a 0.3 m panchromatic band and six spectral bands with a 1.2 m resolution, as well as pansharpened 0.3 m fused multispectral images. Such capabilities promise unprecedented precision in detecting subtle canopy changes indicative of early PWN infestation. However, despite this promising potential, the application of ultra-high-resolution optical imagery for PWN detection remains unexplored, and no study has yet systematically evaluated its performance against established platforms like UAVs.

To address this research gap, the study conducted a comprehensive comparison of ultra-high-resolution BJ3N imagery with high-resolution Beijing 3A satellite (BJ3A) imagery, which has a 0.5 m spatial resolution for the panchromatic band and 2 m for the four multispectral bands, and UAV multispectral imagery with a 0.07 m spatial resolution for five multispectral bands. The objectives are to systematically evaluate the capabilities of ultra-high-resolution remote sensing images and the impact of spatial and spectral resolution on the detection of PWN-infected trees, and to identify the optimal data sources that can balance spatial resolution, spectral capabilities, coverage, and practical applicability, ultimately enhancing the efficiency of future PWN monitoring.

## 2. Materials and Methods

### 2.1. Study Area

**Figure 1:**
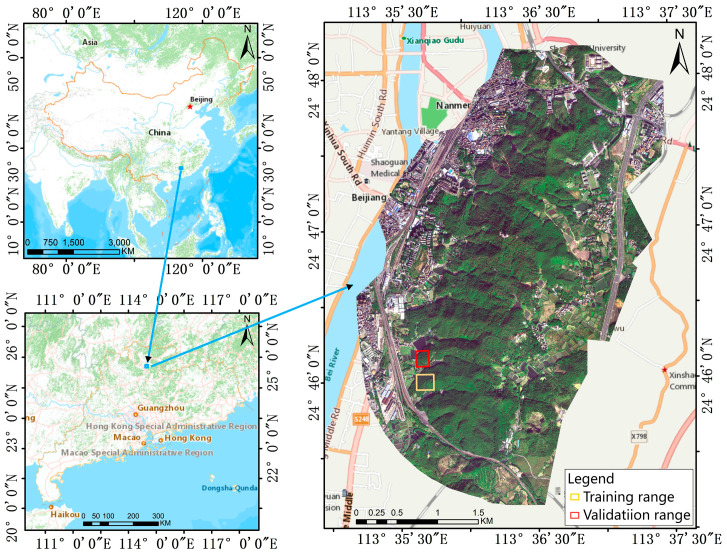
The location of the study area in Shaoguan City, Guandong Province, China.

The study area is located in Shaoguan, Guangdong Province, China (geographical coordinates 113°35′ to 113°37′ E, 24°45′–24°48′ N), as shown in Figure 1. The study area lies within a subtropical mountain monsoon climate zone, characterized by an average annual temperature of approximately 20 °C, an average annual rainfall of 1600 mm, and an annual frost-free period of about 310 days. These climatic conditions are conducive to the proliferation of the pine bark beetle, leading to a particularly severe PWN outbreak in the area. The area covers approximately 5 km^2^, with elevations ranging from 60 to 255 m. Forest coverage reaches around 90%, dominated by species such as horsetail pine, fir, and camphor. A schematic overview of the study area is provided in Figure 1.

### 2.2. Data

The data used in this study included ultra-high-resolution BJ3N satellite imagery and high-resolution Beijing 3A (BJ3A) satellite multispectral imagery, as well as very-high-resolution unmanned aerial vehicle (UAV) multispectral imagery.

#### 2.2.1. BJ3N

The BJ3N satellite was launched on 29 April 2021, as a state-of-the-art, ultra-high-resolution optical remote sensing satellite, jointly developed by China 21st Century Space Technology Co., Ltd. (Beijing, China) and Airbus. It acquires imagery at a spatial resolution of 0.3 m in the panchromatic band and 1.2 m in the multispectral bands, ranking among the highest-resolution commercial remote sensing currently in operation. The main parameters of BJ3N are shown in Table 1.

The imagery of BJ3N for the study area was acquired on 8 December 2021. The processing applied to the BJ3N data included calibration, atmospheric correction, and orthorectification.

To fully leverage the spatial information of BJ3N data and enhance the spatial resolution of its multispectral bands, this study adopts the Gram–Schmidt pansharpening method for data fusion. This approach establishes a transformation by simulating a panchromatic band, enabling the effective incorporation of spatial details while minimizing spectral distortion.

#### 2.2.2. BJ3A

The BJ3A satellite was launched on 11 June 2021 and was developed by China 21st Century Space Technology Co., Ltd. It is known for its “three supers” feature, which includes ultra-high agility, stability, and precision. The satellite is equipped with a high-resolution, wide-swath panchromatic and multispectral bi-directional camera system, providing imagery at spatial resolutions of 0.5 m for the panchromatic band and 2 m for the multispectral bands. The main parameters of BJ3A are shown in Table 2.

The imagery of BJ3A for the study area was acquired on 6 December 2021. The data preprocessing procedure applied to the BJ3A imagery was consistent with that used for the BJ3N data.

#### 2.2.3. UAV

The multispectral UAV imagery for the study area was acquired using a DJI P4 Multispectral RTK (DJI, Shenzhen, China). The UAV is equipped with an integrated MicaSense RedEdge multispectral sensor and a high-precision real-time kinematic (RTK) module, which provides centimeter-level positioning accuracy to support the generation of high-accuracy geotagged images. The sensor captures imagery in five spectral bands within the visible to red-edge and infrared spectrum. The main characteristics of the multispectral sensor are given in Table 3.

The multispectral UAV remote sensing data were acquired on December 18, 2021, at around 11 a.m. local time. The weather conditions during the flight were adequate with enough solar illumination, calm wind with a slight breeze, and no clouds. The raw imagery was processed using DJI Zhitu software 3.0.0, to produce a digital orthophoto map of the study area with a spatial resolution of 0.07 m.

### 2.3. Sample Plotting

The samples were divided into a training set and a validation set, which were collected from two spatially segregated areas within the study area to ensure independence, as illustrated in Figure 1.

#### 2.3.1. Training Set

Pine trees infected with PWN disease typically exhibit visible symptoms. As the infection progresses, the needles of affected trees transition from green to yellowish-brown, then reddish-brown, and eventually dark reddish-brown upon complete death [18]. These color shifts are accompanied by changes in the reflectance of specific wavelength bands, causing the spectral characteristics of the infected trees to deviate from those of healthy vegetation. These spectral changes provide a robust theoretical foundation for detecting PWN-infected trees using optical remote sensing imagery [19]. Notably, healthy pine trees can be easily distinguished from those infected with PWN in the visible light spectrum [20]. Based on these symptomatic color features, this study establishes visual interpretation markers for identifying PWN-infected trees.

Given the diversity of features within the forest area and the influence of light and shadow, this study selects a sub-region with a high concentration of PWN-infected trees as a common training sample area for all three types of remote sensing data. The sample area was characterized by five main feature types: PWN-infected trees, healthy trees, lake, land, and other. Corresponding labels for these features were manually delineated on the BJ3A, BJ3N, and UAV imagery through visual interpretation. These labeled datasets were then used for model training. The image data and corresponding labels for the selected sample areas are presented in Table 4.

#### 2.3.2. Validation Sets

**Figure 2:**
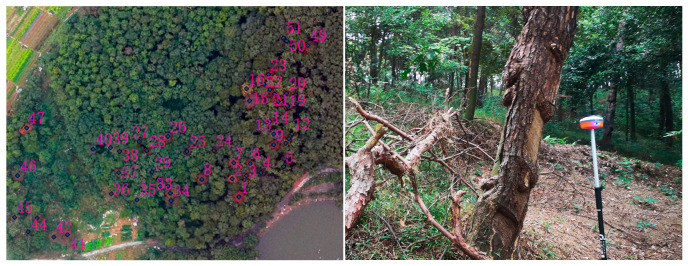
Checkpoint data imagery. The numbers in the figures are the numbers of Validation Sets.

To evaluate the accuracy of the BJ3A, BJ3N, and UAV imagery in detecting PWN-infected trees, a field survey was conducted to collect the geographic coordinates of 51 PWN-infected trees using a GNSS receiver as validation sets (Figure 2).

### 2.4. Research Methodology

#### 2.4.1. U-Net Network Model

Semantic segmentation networks are widely used deep learning models for identifying PWN-infected trees in high-resolution remote sensing imagery [21,22,23]. Previous studies have demonstrated the effectiveness of U-Net in this context. For example, Ye et al. [23] compared the performance of U-Net and SVM algorithms for identifying PWN-infected trees using Landsat imagery, with U-Net achieving an accuracy of 0.60, compared to 0.21 for SVM. Similarly, Han et al. [24] evaluated several deep learning algorithms and found that the U-Net model yielded the highest accuracy for detecting PWN outbreaks in high-resolution imagery. Based on these findings, the U-Net model was selected as the most suitable approach for extracting PWN-infected trees in this study.

The U-Net network model [25], initially introduced in 2015 at the International Conference on Medical Image Computing and Computer-Assisted Intervention (MICCAI), is a classic architecture for semantic segmentation. It builds upon the Fully Convolutional Network (FCN) by incorporating convolutional layers, max-pooling layers, inverse convolutional layers, and ReLU nonlinear activation functions. It utilizes a symmetric U-shaped architecture, consisting of two main components: the encoder and the decoder. The encoder, situated on the left, performs downsampling to extract imagery details, whereas the decoder on the right executes upsampling to restore these details, thus enabling the accurate localization of the target [25]. This model exhibits strong segmentation capabilities, effectively fusing both high- and low-dimensional information. It can be trained with relatively limited data, yielding a robust model for edge extraction [26]. The network ultimately produces a feature map with the same resolution as the input image.

#### 2.4.2. Accuracy Evaluation Method

To evaluate model performance, this study employs precision (P), recall (R), and the F1 score [27]. These accuracy metrics are defined as follows:

(1) $$P=\frac{TP}{TP+FP}$$

(2) $$R=\frac{TP}{TP+FN}$$

(3) $$F1=\frac{2P\times R}{P+R}$$

where TP denotes the number of correctly identified infected trees, FP denotes the number of falsely identified trees, and FN denotes the number of undetected infected trees.

P, also known as the positive predictive value, reflects the proportion of true positives among all predicted positives, indicating the accuracy of the prediction model. A higher P value suggests a greater likelihood of correct predictions, and consequently, better model performance. R, or sensitivity, measures the proportion of actual infected trees correctly identified by the model. A higher R value indicates a better ability to correctly predict the true positives. The F1 score is the harmonic mean of precision and recall, balancing the tradeoff between the two metrics. Since precision and recall are often inversely related, improving one typically sacrifices the other. Thus, the F1 score provides a comprehensive measure of model performance, where a higher value of F1 indicates an optimal balance between precision and recall, reflecting a more accurate and reliable model.

## 3. Results and Analyses

### 3.1. Experimental Setup

The U-Net network model was conducted in Matlab software R2023b, on a computer equipped with an AMD Ryzen 7 5800H CPU, 16 GB of memory, and an NVIDIA GeForce RTX 3050 Ti graphics card. A set of training options was configured through programming, and the Stochastic Gradient Descent with Momentum (SGDM) optimization algorithm was used for training with momentum. During the training process, the base learning rate was set to 0.01, with 2000 iterations per epoch. Each iteration used a mini-batch size of 8, and MaxEpochs was set to 1. The BJ3A, BJ3N, and UAV image data were paired with the corresponding sample training set. In each iteration of every training epoch, mini-batches consisting of eight image patches of size 128 × 128 pixels were fed into the network. To avoid excessive memory usage by large images and effectively augment the available training data, 16,000 mini-batches were processed per epoch.

### 3.2. Identifying PWN-Infected Trees Using BJ3N Imagery

#### 3.2.1. Using Multispectral Imagery with Raw Spatial Resolution of 1.2 m

The BJ3N satellite image comprises six raw multispectral bands at a resolution of 1.2 m, including red (R), green (G), blue (B), near-infrared (NIR), red-edge, and deep-blue. Four different band combinations were sequentially used as input to the training samples: the three-band (R, G, B), four-band (R, G, B, NIR), five-band (R, G, B, NIR, Red-edge), and six-band (R, G, B, NIR, Red-edge, Deep-blue) combinations. For each combination, a corresponding U-Net model was constructed under the specified training environment and parameters, incorporating the respective labeled data. The models’ accuracies were evaluated using validation sets, as presented in Table 5.

**Figure 3:**
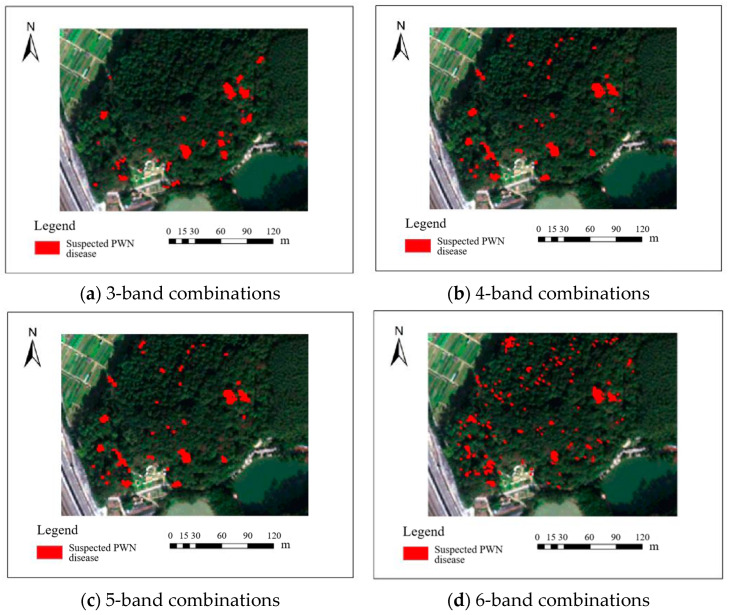
Results of BJ3N satellite with 0.5 m raw spatial resolution for identifying PWN-infected trees in this study area.

Four raw band combinations of the BJ3N satellite at 1.2 m resolution showed low accuracy in identifying PWN-infected trees, and these identification results are illustrated in Figure 3. The three-band combination with red (R), green (G), and blue (B) achieved the highest F1 score of 66.6%. The four-band combination (R, G, B, and NIR) and the five-band combination (R, G, B, NIR, and Red-edge) yielded F1 scores of 50.5% and 50.4%, respectively, while the six-band combination (R, G, B, NIR, Red-edge, and Deep-blue) performed the worst, with an F1 score of only 24%.

#### 3.2.2. Using Multispectral Imagery with a Fused Spatial Resolution of 0.3 m

The BJ3N satellite’s 0.3 m panchromatic band and six 1.2 m multispectral bands were fused using the pansharpening fusion method to generate six fused multispectral bands at a spatial resolution of 0.3 m. Like raw spatial resolution multispectral imagery, the fused three-band (R, G, B), fused four-band (R, G, B, NIR), fused five-band (R, G, B, NIR, Red-edge), and fused six-band (R, G, B, NIR, Red-edge, Deep-blue) combinations were subsequently employed as input to the training samples for constructing U-Net models to identify PWN-infected trees. The models were trained under the predefined environment and parameters, incorporating the corresponding labeled data. The models’ accuracy was evaluated using validation sets, as presented in Table 5.

**Figure 4:**
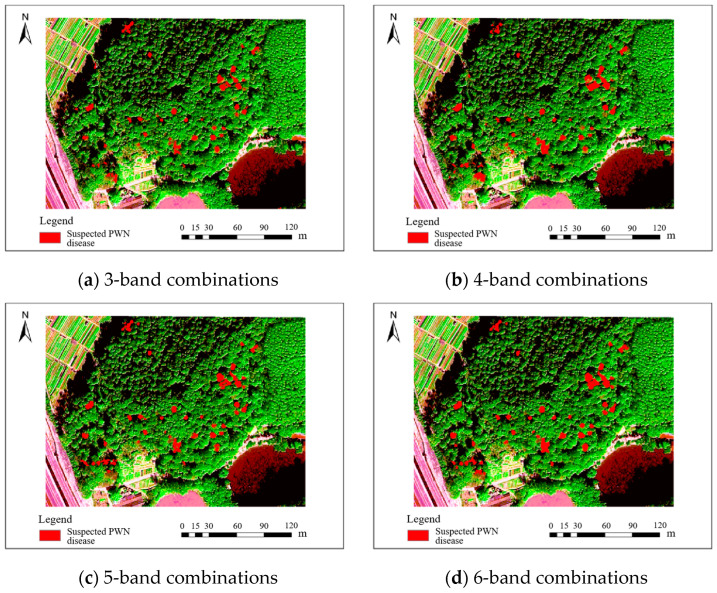
Results of BJ3N satellite with 0.3 m fused spatial resolution for identifying PWN-infected trees in this study area.

The accuracy of identifying PWN-infected trees using the fused 0.3 m BJ3N multispectral imagery was significantly higher than that achieved with the raw 1.2 m data, with only minor differences observed among the various band combinations. Among these, the fused five-band combination (R, G, B, NIR, Red-edge) achieved the highest F1 value of 88.9%, followed by the fused three-band (R, G, B) combination with an F1 value of 85.1%, and the fused six-band (R, G, B, NIR, Red-edge, Deep-blue) combination with an F1 value of 84.3%. The fused four-band (R, G, B, NIR) combination demonstrated the lowest precision, with an F1 value of 81.9%. All these identification results are illustrated in Figure 4.

### 3.3. Identifying PWN-Infected Trees Using BJ3A Imagery

#### 3.3.1. Using Multispectral Imagery with a Raw Spatial Resolution of 2 m

The BJ3A satellite image comprises four raw multispectral bands with a spatial resolution of 2 m, including R, G, B, and NIR. During the training process, three-band combinations (R/G/B) and four-band combinations (R/G/B/NIR) serve as input variables for the U-Net model. The model was built using the predefined training environment and parameters, along with the corresponding labeled data. The models’ accuracy was evaluated using the test samples, as summarized in Table 6.

**Figure 5:**
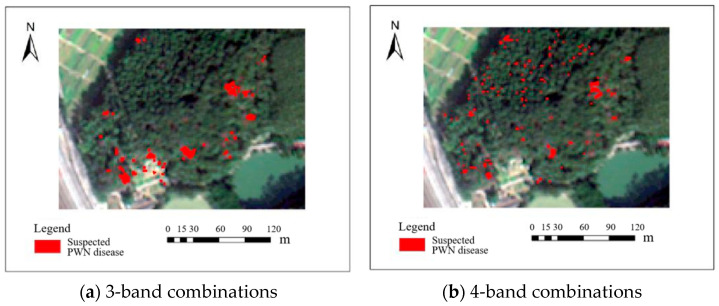
Results of the BJ3A satellite with 2 m raw spatial resolution for identifying PWN-infected trees in this study area.

Two raw band combinations of the BJ3A satellite at 2 m resolution showed limited accuracy in identifying PWN-infected trees, with F1 scores of 58% for the three-band (R, G, B) and 28% for the four-band (R, G, B, NIR) combination. All these identification results are illustrated in Figure 5.

#### 3.3.2. Using Multispectral Imagery with a Fused Spatial Resolution of 0.5 m

**Figure 6:**
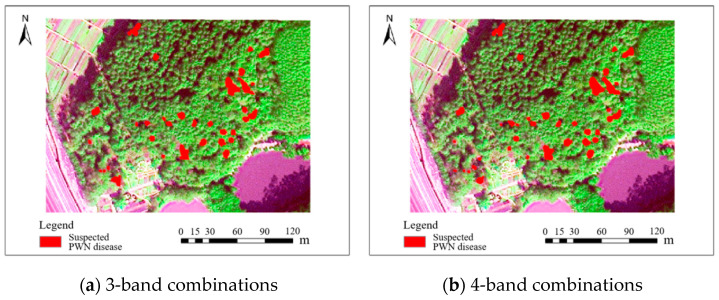
Results of the BJ3A satellite with 0.5 m fused spatial resolution for identifying PWN-infected trees in this study area.

The pansharpening fusion technique was applied to combine the 0.5 m panchromatic band with the four 2 m multispectral bands from the BJ3A satellite, resulting in four fused multispectral bands at 0.5 m resolution. The fused three-band (R, G, B) and fused four-band (R, G, B, NIR) combinations were subsequently employed as input to the training samples for constructing U-Net models to identify PWN-infected trees. The models were trained under the predefined environment and parameters, incorporating the corresponding labeled data. The models’ accuracy was evaluated using validation sets, as presented in Table 6, and the identification results are illustrated in Figure 6.

The accuracy of identifying PWN-infected trees was significantly improved by using 0.5 m fused band combinations of the BJ3A satellite, compared with the raw band combinations. Notably, the F1 score for the fused three-band combination of R, G, and B reached 86.3%. The fused four-band combination of R, G, B, and NIR exhibited a similar performance, with an F1 score of 85.8%, indicating comparable performance between the two band combinations in identifying PWN-infected trees.

### 3.4. Identifying PWN-Infected Trees Using UAV Multispectral Imagery

The UAV multispectral imagery used in this study has five spectral bands: R, G, B, NIR, and Red-edge. The three-band (R, G, B), four-band (R, G, B, NIR), and five-band (R, G, B, NIR, Red-edge) combinations were subsequently employed as input to the training samples for constructing U-Net models to identify PWN-infected trees. The models were trained under the predefined environment and parameters, incorporating the corresponding labeled data. The accuracy of each model was assessed based on the validation sets, as summarized in Table 7.

**Figure 7:**
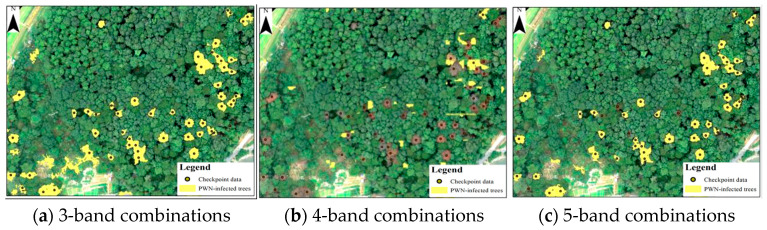
Results of UAV multispectral bands with a spatial resolution of 0.7 m for identifying PWN-infected trees in this study area.

Comparison with BJ3N and BJ3A satellite imagery, 0.07 m UAV multispectral imagery achieved the highest accuracy in identifying PWN-infected trees. Among UAV band combinations, there were few differences in the identification accuracy. The five-band combinations of R, G, B, NIR, and Red-edge demonstrated the highest accuracy, achieving an F1 score of 89.1%. Both the four-band combinations of R, G, B, and NIR and the three-band combinations of R, G, and B exhibited similar performance, with F1 scores of 87.7% and 88.5%, respectively. All these identification results are illustrated in Figure 7.

## 4. Discussion

### 4.1. Effect of Spatial Resolution of Remote Sensing Images on Identification of PWN-Infected Trees

When comparing the F1 accuracy of BJ3N, BJ3A, and UAV band combination in identifying PWN-infected trees separately, it was found that there was a clear relationship between spatial resolution and identifying accuracy. The UAV imagery at 0.07 m resolution achieved the highest F1 score of 89.1%, followed by the fused BJ3N imagery at 0.3 m and the fused BJ3A imagery at 0.5 m, with F1 scores of 88.9% and 88.5%, respectively. In comparison, the raw-resolution BJ3N imagery at 1.2 m and raw-resolution BJ3A imagery at 2.0 m attained maximum F1 scores of only 66.6% and 58.0%, respectively. These results demonstrate that higher spatial resolution in remote sensing imagery leads to improved accuracy in identifying PWN-infected trees. Ota et al. [28] also found that spatial resolution has a certain impact on tree species identification. The accuracy of identifying tree species using 4 m high-resolution remote sensing images is significantly higher than that using 30 m coarse resolution images. However, there is not much difference in the accuracy of identifying tree species using 25 m and 30 m resolution remote sensing images. Similarly, Zhang et al. [10] examined a sample set of semantic segmentation model input sizes and found that increasing the resolution of images enhances the recognition accuracy of each category of the semantic segmentation model, further supporting the importance of spatial resolution in remote sensing-based detection tasks.

Bolch et al. [29] demonstrated that increasing the spatial resolution of hyperspectral UAV imagery improved the detection accuracy of small invasive aquatic plant patches, though at a significantly higher cost, highlighting the need to balance detection accuracy with resolution in practical applications. Similarly, Saltiel et al. [30] discovered that enhancing image spatial resolution from 22.8 cm to 7.8 cm only marginally improved wetland vegetation recognition accuracy using a deep learning semantic segmentation model, while substantially increasing processing time and reducing coverage. These findings align with the study, in which the F1 accuracy for identifying PWN-infected trees using fused 0.3 m BJ3N multispectral imagery differed by only 0.2% from that achieved with 0.07 m UAV multispectral imagery.

In contrast, Müllerová et al. [31] resampled 0.05 m UAV imagery into 0.5 m Pleiades remote sensing image data and found that spatial resolution had little effect on the identification of tree species. However, the study reveals that for the interval of spatial resolution between 0.07 m UAV images and 0.5 m satellite remote sensing images, there is a certain impact on recognizing tree species, with the F1 value decreasing by 2.8%.

Based on the above discussion, it is evident that 0.3 m ultra-high-resolution may represent the optimal choice for detecting PWN-infected trees in a subtropical mountain monsoon climate zone.

### 4.2. Effect of Spectral Features of Remote Sensing Imagery on Identification of PWN-Infected Trees

Based on the analysis of 0.07 m UAV imagery, the identification accuracy achieved with the three-band combination (R, G, B) was comparable to that of the four-band and five-band combinations across precision, recall, and F1-score metrics. A similar pattern was observed for both BJ3N and BJ3A imagery, where no significant differences in identification accuracy were found among the various band combinations at same spatial resolutions. Collectively, these findings indicate that the number of spectral bands has a negligible impact on the accuracy of identifying PWN-infected trees, and that a limited number of bands can be sufficient to achieve high performance.

This phenomenon is supported by Lopatin et al. [32], who compared UAV RGB and hyperspectral imagery for detecting three invasive species in the forests of south-central Chile. Their study demonstrated that the recognition accuracy of RGB imagery using only three bands exceeded that of hyperspectral imagery with 41 bands for two of the invasive species. This result challenges the conventional assumption that a higher number of spectral bands necessarily leads to improved classification accuracy, suggesting instead that increasing spectral resolution does not always enhance species identification outcomes. Therefore, the number of spectral features is not a critical factor in identifying PWN-infected trees, and a three-band combination (R, G, B) can be sufficient for effective detection using high-resolution remote sensing imagery.

## 5. Conclusions

This study investigates the identification of PWN-infected trees using ultra-high-resolution optical remote sensing satellite images. By comparing the accuracy of identifying PWN-infected trees using BJ3N, as well as BJ3N and UAV imagery, it was found that the higher the spatial resolution, the higher the accuracy of identifying PWN-infected trees. The significance of spectral features in identifying PWN-infected trees varies among different spatial resolution remote sensing images. Nevertheless, the impact of differences in spectral resolution diminishes for identifying PWN-infected trees at higher spatial resolutions. The ultra-high-resolution BJ3N remote sensing imagery with a spatial resolution of 0.3 m exhibited lower false detection and missed detection rates when identifying trees affected by pine wilt nematode (PWN), indicating that a spatial resolution of 0.3 m is sufficient for detecting PWN-affected trees and is the closest to the resolution saturation point in a subtropical mountain monsoon climate zone. Therefore, ultra-high-resolution optical remote sensing satellite imagery clearly demonstrates the optimal resolution for detecting PWN-infected trees, while also offering advantages such as repeatable data acquisition and extensive spatial coverage. Moreover, ultra-high-resolution remote sensing can be applied to forest disease monitoring and prevention strategies to enhance the practical value of research.

## Figures and Tables

**Table 1:** BJ3N satellite parameters.

Category	Parameter	
Satellite Orbit	Sun-synchronous orbit	
Orbit Altitude	620 km	
Spatial Resolution	Panchromatic	0.3 m
	Multispectral	1.2 m
Spectral Bands	Panchromatic	450–800 nm
	Multispectral	Deep Blue: 400–450 nm
		Blue: 450–520 nm
		Green: 530–590 nm
		Red: 620–690 nm
		Red Edge: 700–750 nm
		Near-Infrared: 770–880 nm

**Table 2:** BJ3A satellite parameters.

Category	Parameter	
Satellite Orbit	Sun-synchronous orbit	
Orbit Altitude	500 km	
Spatial Resolution	Panchromatic	0.5 m
	Multispectral	2.0 m
Spectral Bands	Panchromatic	450–700 nm
	Multispectral	Blue: 450–520 nm
		Green: 520–590 nm
		Red: 630–690 nm
		Near-Infrared: 770–890 nm

**Table 3:** Characteristics of the multispectral sensor.

Category	Parameter	
Lens	FOV: 62.7°; Focal length: 5.74 mm; Fixed focus at infinity; Aperture: f/2.2	
Imaging Sensor	1/2.9inch CMOS	Including 1 color sensor for visible light imaging and 5 monochrome sensors for multispectral imaging
	Individual Sensor	Effective pixels: 2.08 million (total pixels: 2.12 million)
Spectral bands	Blue: 434–466 nm	
	Green: 544–576 nm	
	Red: 634–666 nm	
	Red Edge: 714–746 nm	
	Near-Infrared: 814–866 nm	

**Table 4:** Sample area and label drawing results of BJ3A images, BJ3N images, and UAV imagery.

Image Type	Sample Area	Label Drawing Results
BJ3A imagery	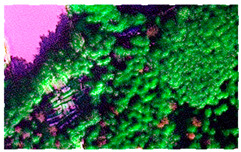	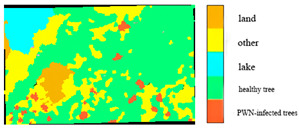
BJ3N imagery	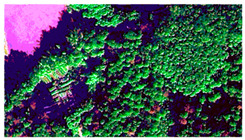	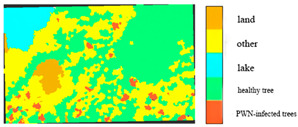
UAV imagery	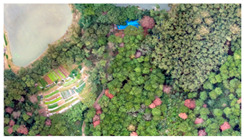	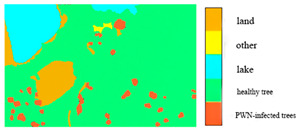

Note: The sample labels have been categorized into five groups: land is represented by yellow, other features by orange, lakes by blue, healthy trees by green, and PWN-infected trees by red.

**Table 5:** The accuracy of identifying PWN-infected trees using the BJ3N satellite’s multispectral bands with a raw spatial resolution of 1.2 m.

Image Type	Resolution/m	Band Type	P/%	R/%	F1/%
raw spatial resolution	1.2	R, G, B, NIR, Red-edge, Deep-blue	25.8	22.5	24
		R, G, B, NIR, Red-edge	41.3	64.7	50.4
		R, G, B, NIR	52.1	49	50.5
		R, G, B	71.1	62.7	66.6
fused spatial resolution	0.3	R, G, B, NIR, Red-edge, Deep-blue	84.3	84.3	84.3
		R, G, B, NIR, Red-edge	91.7	86.3	88.9
		R, G, B, NIR	82.7	81.1	81.9
		R, G, B	86	84.3	85.1

**Table 6:** The accuracy of identifying PWN-infected trees using the BJ3A satellite’s multispectral bands with a raw spatial resolution of 2 m.

Image Type	Resolution/m	Band Type	P/%	R/%	F1/%
raw spatial resolution	2	R, G, B, NIR	22.4	37.3	28
		R, G, B	64.3	52.9	58
fused spatial resolution	0.5	R, G, B, NIR	89.4	82.4	85.8
		R, G, B	93.2	80.4	86.3

**Table 7:** The accuracy of identifying PWN-infected trees using the UAV multispectral bands with a spatial resolution of 0.7 m.

Image Type	Resolution/m	Band Type	P/%	R/%	F1/%
UAV multispectral imagery	0.07	R, G, B, NIR, Red-edge	83.1	96.1	89.1
		R, G, B, NIR	79.4	98	87.7
		R, G, B	80.6	98	88.5

## Data Availability

The data supporting the study findings are available on request from the corresponding author. The data are not publicly available due to the authors’ plan to conduct a series of follow-up studies based on this dataset.

## Abbreviations

The following abbreviations are used in this manuscript:

PWN	Pine wood nematode
BJ3N	Beijing 3 International Cooperative Remote Sensing Satellite
BJ3A	Beijing 3A satellite
UAV	Unmanned aerial vehicle
ANNs	artificial neural networks
SGDM	Stochastic Gradient Descent with Momentum
